# Revision of Australian Matini diving beetles based on morphological and molecular data (Coleoptera, Dytiscidae, Matinae), with description of a new species

**DOI:** 10.3897/zookeys.293.4472

**Published:** 2013-04-19

**Authors:** Lars Hendrich, Michael Balke

**Affiliations:** 1 Zoologische Staatssammlung, Münchhausenstraße 21, D-81247 München, Germany

**Keywords:** *Allomatus*, *Batrachomatus*, new species, synonymy, morphological characters, molecular taxonomy

## Abstract

Morphological characters and mitochondrial DNA sequence data were used to revise the Australian diving beetles in the genera *Allomatus* Mouchamps, 1964 and *Batrachomatus* Clark, 1863. As a result of these studies *Allomatus* **syn. n.** is synonymised with *Batrachomatus*, and *Allomatus nannup* Watts, 1978 from SW Australia and *Allomatus wilsoni* Mouchamps, 1964 from SE Victoria are transferred to *Batrachomatus*. The four Australian Matini species knownso far are re-described, and *Batrachomatus larsoni* **sp. n.** from the Windsor Tableland in NE Queensland is described. After more than 40 years *Batrachomatus wilsoni* has been re-discovered in two rivers in Victoria. We delineate the species using traditionally employed morphological structures such as in the male genitalia and body size, shape and colour pattern, as well as mitochondrial *cox1* sequence data for 20 individuals. Important species characters (median lobes, parameres and colour patterns) were illustrated. We provide an identification key and outline distribution and habitat preferences of each species. All Australian Matini are lotic, inhabiting permanent and intermittent streams, creeks and rivers.

## Introduction

The Matini, the single tribe of the subfamily Matinae, is thus far known to contain eight species in three genera. Their current distribution is highly disjunct. *Matus* Aubé, 1836 is distributed in the Nearctic region with four species and one subspecies. In Australia there are two species so far assigned to *Allomatus* Mouchamps, 1964 and two assigned to *Batrachomatus* Clark, 1863. The American species were revised by Larson et al. (2000), and the Australian ones by Mouchamps (1964) and Watts (1978). The larvae of *Allomatus nannup*, *Batrachomatus daemeli*, *Matus bicarinatus* (Say, 1823) and *Matus leechi* Young, 1953 have been described and phylogenetic relationships within the Matini based on larval characters have been discussed by Wolfe and Roughley (1985), Alarie et al. (2001) and Alarie and Butera (2003).

Despite their disjunct distribution, members of the Matinae are postulated to share a monophyletic origin and to be relicts of a once more extensively distributed taxon (Alarie et al. 2001, Alarie and Butera 2003).

The aim of this paper is to revise the Australian Matini taxonomically, combining morphology and mitochondrial DNA sequence data. *Allomatus* syn. n. issynonymized with *Batrachomatus*, we describe one new species from the Atherton Tableland in Queensland and provide a key to the five Australian species known so far. All DNA sequence data and digital images of morphological structures were also made available online in Wiki format for faster dissemination of taxonomic knowledge and the option of future edits and additions to the species pages in a fully versioned framework.

The two species of the genus *Batrachomatus* are widespread in tropical northern Australia (*Batrachomatus wingii*) and in south-eastern Australia (*Batrachomatus daemeli*), whereas all species of the former genus *Allomatus* are restricted to one or two river systems in the southwest (*Allomatus nannup*), or the southeast (*Allomatus wilsoni*). Except for *Allomatus nannup* and *Batrachomatus daemeli*, all other species are very rarely collected and are mainly known from only a few specimens from their type localities. One undescribed species from northern Queensland was found among the Dytiscidae donated by D.J. Larson (Maple Creek, Canada) to the Australian National Insect Collection (ANIC) in Canberra.

## Material and Methods

**Material**: We examined 401 specimens, including the type material of all species.

**Descriptions**: Beetles were studied with a Leica MZ 12.5 microscope at 10–100x. Photos of the male genitalia, used for the drawings, were made using a digital photo imaging system, composed of a Leica DM 2500 M microscope and a Tucsen 5.0 MP camera. This microscope was fitted with Leica HCX PL “Fluotar” 5x and 10x metallurgical grade lenses (Buffington and Gates 2008). Image stacks were aligned and assembled with the computer software Helicon Focus 4.77^TM^. The drawings were scanned and edited, using the software Adobe Illustrator CS5.1. Label data of type material were cited in quotation marks. All type specimens of the herein described species were provided with red labels.

The terminology to denote the orientation of the genitalia follows Miller and Nilsson (2003). The following abbreviations were used: TL (total length), TL-H (total length without head), and MW (maximum width).

**Coordinates** are given in decimal notation unless cited verbatim from labels. Beside various Australian road maps, we also used Google Earth (http://earth.google.com) to locate localities.

**DNA sequencing and data analysis**:The sequence data originate from Hendrich et al. (2010). We preserved a part of our collections in 96% ethanol and later extracted DNA for sequencing. The laboratory methods employed are detailed on our DNA laboratory wiki: http://zsm-entomology.de/wiki/The_Beetle_D_N_A_Lab DNA Lab. PCR conditions with Mango Taq (Bioline) were 1’ 94°C – 40x (30s 94°C – 30s 47°C – 1’ 72°C) – 10’ 72°C – (hold at 14°C) with primers Jerry and Pat to amplify and sequence the 3’ of the gene encoding for cytochrome *c* oxidase 1 (Simon et al. 1994). Individual beetles from which we extracted and sequenced DNA all bear a green cardboard label that indicates the DNA extraction number of M. Balke (e.g. “DNA M. Balke 2775”). This number links the DNA sample, the dried mounted voucher specimen, deposited in ZSM and GenBank entries (FR733184, FR733183, AY138729, FR733508, FR733182, FR733180, FR732763, FR733507, FR733506, HF912239, HF912240, FR733181, FR732655, AY138730, FR733515, FR733517, FR733516, FR732697, FR733514, FR733513, FR732698, HF912238). We use GARLI V.0.951 (Zwickl 2006) with default settings (using the GTR model of evolution with parameter estimation) to obtain a maximum likelihood tree based on our cox1 data, node confidence was evaluated using the same program and 100 bootstrap replications.

### Codens

**AMS** Australian Museum Sydney, New South Wales, Australia

**ANIC** Australian National Insect Collection, Canberra, Australia

**BMNH** The Natural History Museum, London, UK

**CGC** Collection Gilbert L. Challet, Florida, United States

**CLH** Collection Lars Hendrich, Munich, Germany; property of the NMW

**MVMA** Museum of Victoria, Melbourne, Victoria, Australia

**NMW** Naturhistorisches Museum Wien, Austria

**SAMA** South Australian Museum, Adelaide, South Australia, Australia

**HNHM** Hungarian Natural History Museum,Budapest, Hungary

**ZSM** Zoologische Staatssammlung München, Munich, Germany.

### Collecting procedures

All of the specimens collected by the senior author were obtained by using a strong aquatic Kick Sampling Net with a long pole. Mesh diameters varied from 0.5 to 1.0 mm. Submerged roots and vegetation, stones and rotten logs were swept heavily; the material obtained was then placed on a white nylon sheet (1 m²) or in a white plastic box. Specimens were collected with forceps or by hand.

## Taxonomy

### Batrachomatus

Clark, 1863

http://species-id.net/wiki/Batrachomatus

Batrachomatus Clark, 1863: 15 (type species *Batrachomatus wingii* Clark, 1863, by monotypy); Zimmermann 1919: 215 (cat.); Mouchamps 1964: 137 (descr.); Watts 1978: 119 (descr.); Watts 1985: 23 (cat.); Lawrence et al. 1987: 351 (cat.); Nilsson 2001: 261 (cat.); Larson 1993: 50 (cat.); Watts 2002: 31, 46 (cat.).
Allomatus Mouchamps, 1964: 137 (type species *Allomatus wilsoni* Mouchamps, 1964, by original designation); Watts 1978: 117 (descr.); Watts 1985: 23 (cat.); Lawrence et al. 1987: 351 (cat.); Nilsson 2001: 261 (cat.); Watts 2002: 30, 46 (cat.); **syn. n.**

#### Remarks.

Medium-sized (TL = 6.9–9.6 mm), elongate, shiny and flattened mainly black diving beetles, unicolorous or with testaceous or ferruginous markings on elytra. Epipleuron in apical half more than twice as wide as base of longer spur of metatibia. Both parameres abruptly narrowed near middle. Outer metatarsal claw curved, prosternum with median furrow. Body covered with dense fine punctures and/or very fine microreticulation, meshes irregular, polygonal.

*Allomatus* was justified as a genus based only on the presence of a reticulated pronotum and elytra without any punctuation in contrast to *Batrachomatus* which has a non-reticulate but densely and finely punctate pronotum and elytra (Mouchamps 1964, Watts 1978). *Brachomatus larsoni* sp. n. has characters from both genera: a polygonal double reticulation with punctures at the intersections of all meshes.

The *cox1* sequence data show (Fig. 15) that *Allomatus wilsoni* is sister to *Batrachomatus daemeli*, *Batrachomatus wingi* sister to these two, and *Allomatus nannup* sister to all of these. We use *Matus bicarinatus* (Say, 1823) from North America as well as *Hygrobia maculata* Britton, 1981 and *Hygrobia wattsi* Hendrich, 2001 as outgroups (*Hygrobia* were pruned for Fig. 15). Consequently *Allomatus* Mouchamps, 1964 is here synonymised with *Batrachomatus* Clark, 1863.

### Species checklist

Abbreviations: NSW = New South Wales, QLD = Queensland, TAS = Tasmania, VIC = Victoria.

*Batrachomatus daemeli* (Sharp, 1882) NSW, VIC, TAS

*Batrachomatus larsoni* **sp. n. **N QLD

*Batrachomatus nannup* (Watts, 1978) **comb. n.** SW Australia (Blackwood River)

*Batrachomatus wilsoni* (Mouchamps, 1964) **comb. n.** S VIC, S NSW

*Batrachomatus wingii* Clark, 1863 N WA, NT, QLD

For detailed distribution, see also Figs 13, 14.

**Figures 1–4.**
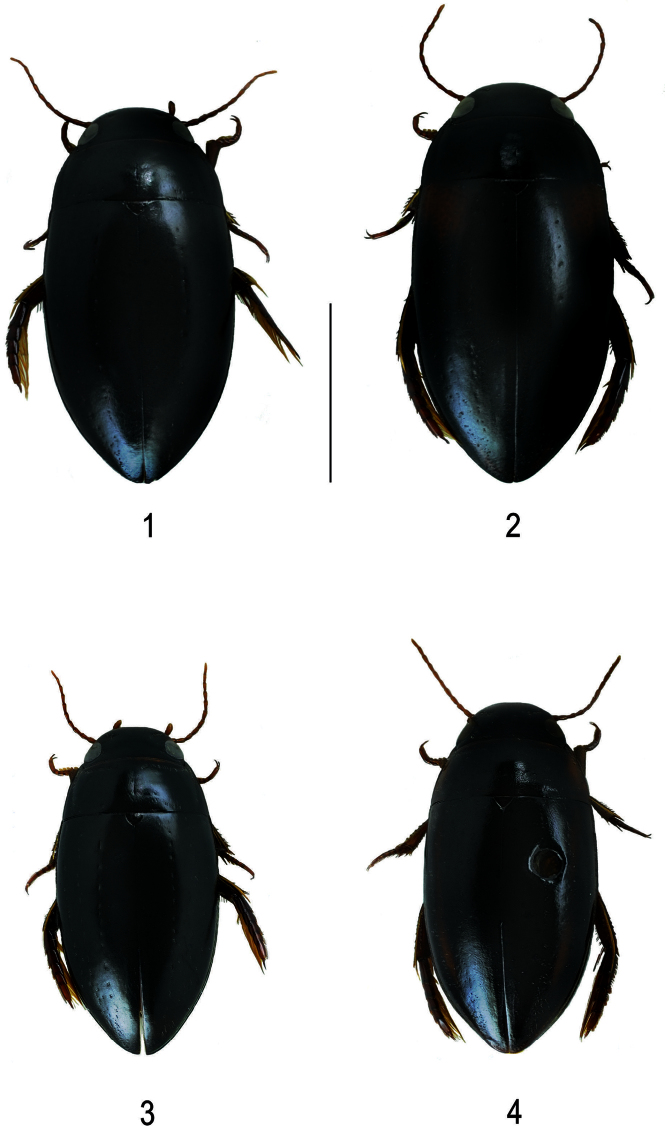
Habitus of **1** *Batrachomatus daemeli* (black form) **2** *Batrachomatus daemeli* (reddish shoulders) **3** *Batrachomatus daemeli* (small form) **4** *Batrachomatus larsoni* sp. n. (paratype) (scale bar = 4 mm).

**Figures 5–7.**
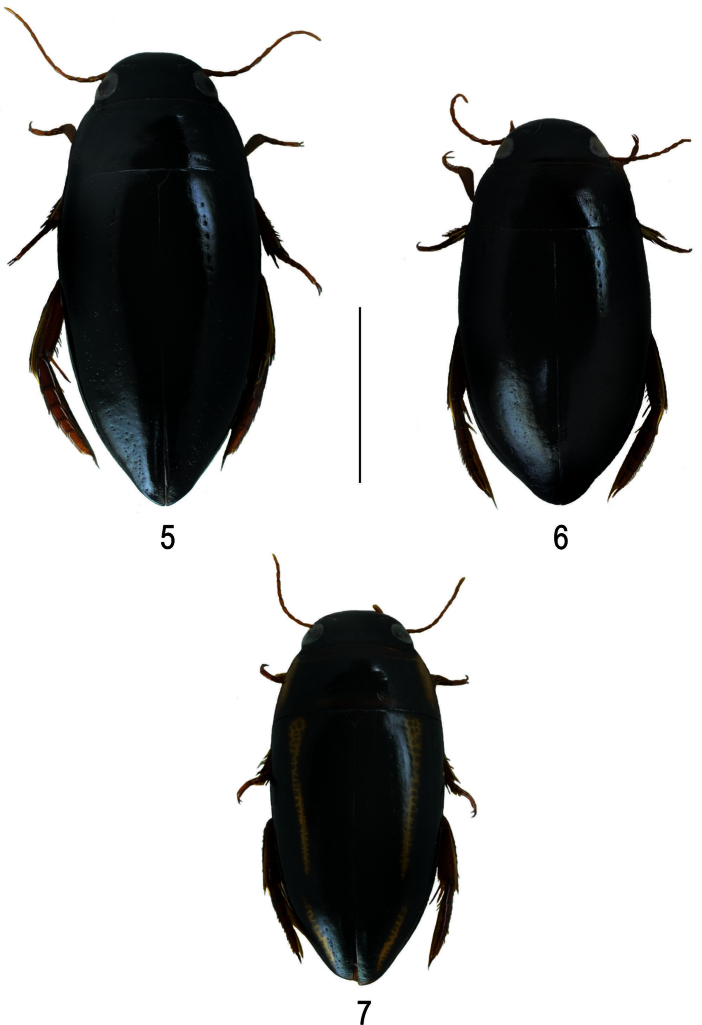
Habitus of **5** *Batrachomatus nannup* **6** *Batrachomatus wilsoni* **7** *Batrachomatus wingii* (scale bar = 4 mm).

**Figures 8–10.**
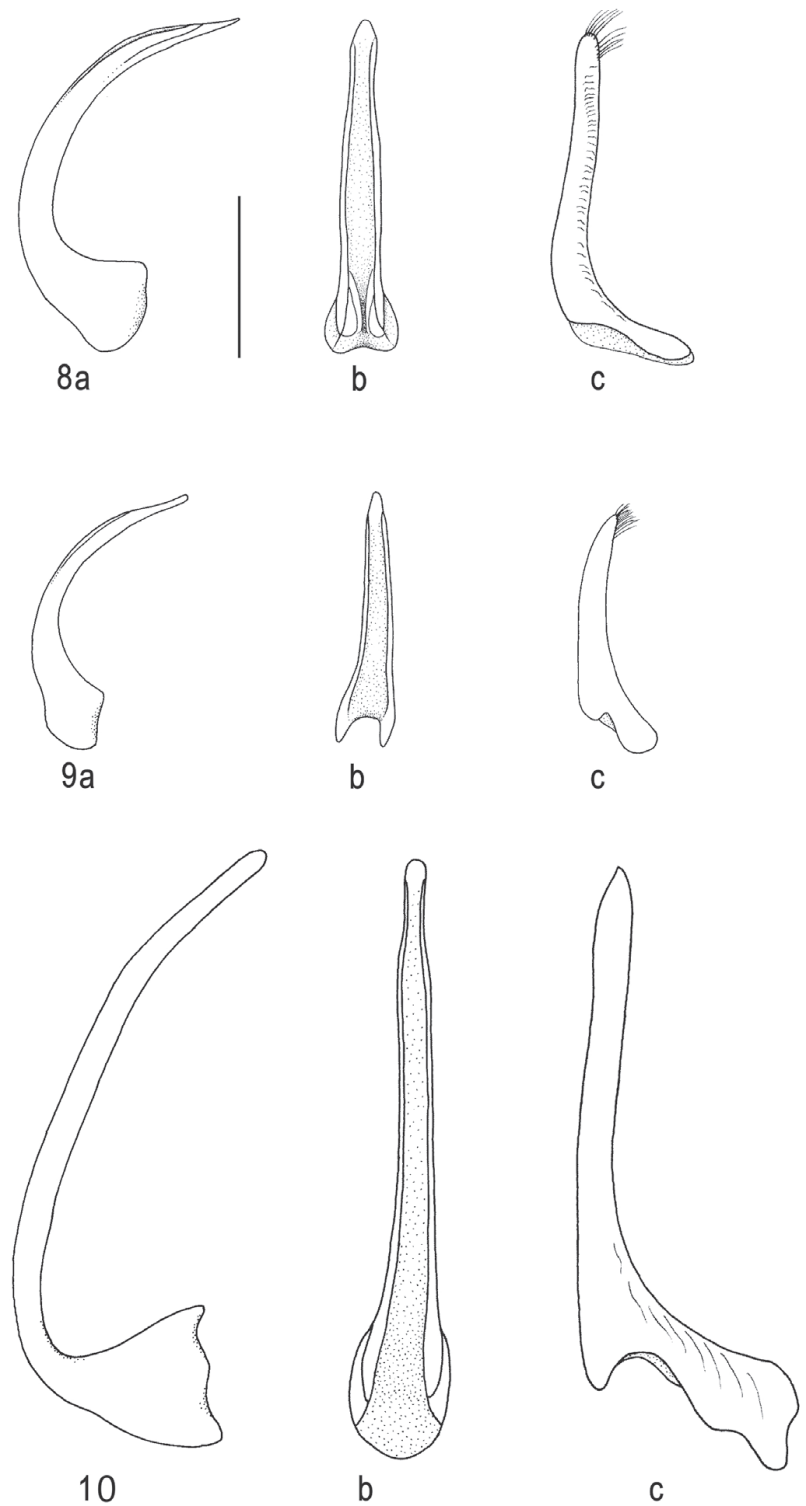
Median lobe of aedeagus in ventral (**a**) and lateral view (**b**), and right paramere in lateral view (**c**): **8** *Batrachomatus daemeli* **9** *Batrachomatus larsoni* sp. n. **10** *Batrachomatus nannup* (scale bar = 0.5 mm).

**Figures 11–12.**
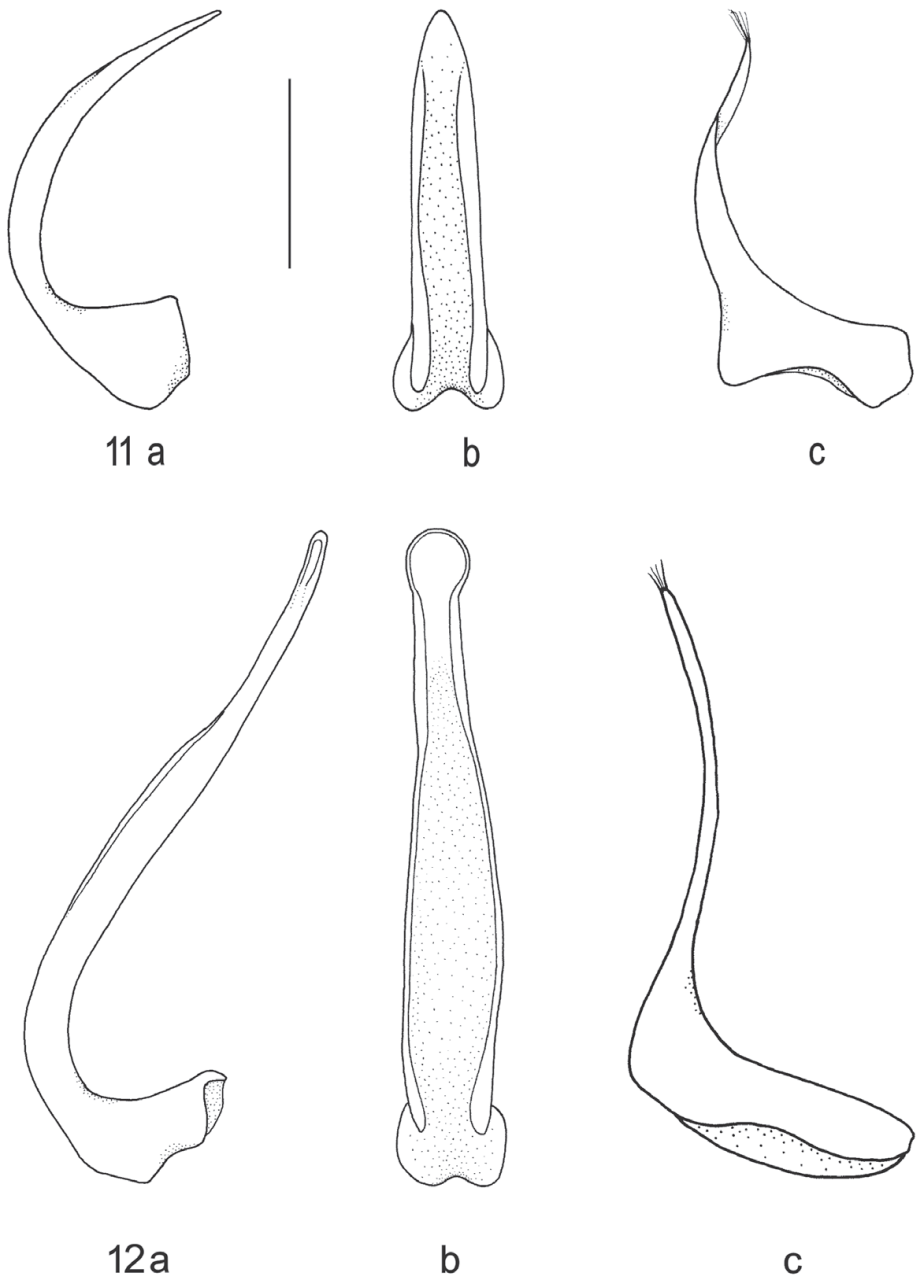
Median lobe of aedeagus in ventral (**a**) and lateral view (**b**), and right paramere in lateral view (**c**): **11** *Batrachomatus wilsoni* **12** *Batrachomatus wingii* (scale bar = 0.5 mm).

**Figure 13.**
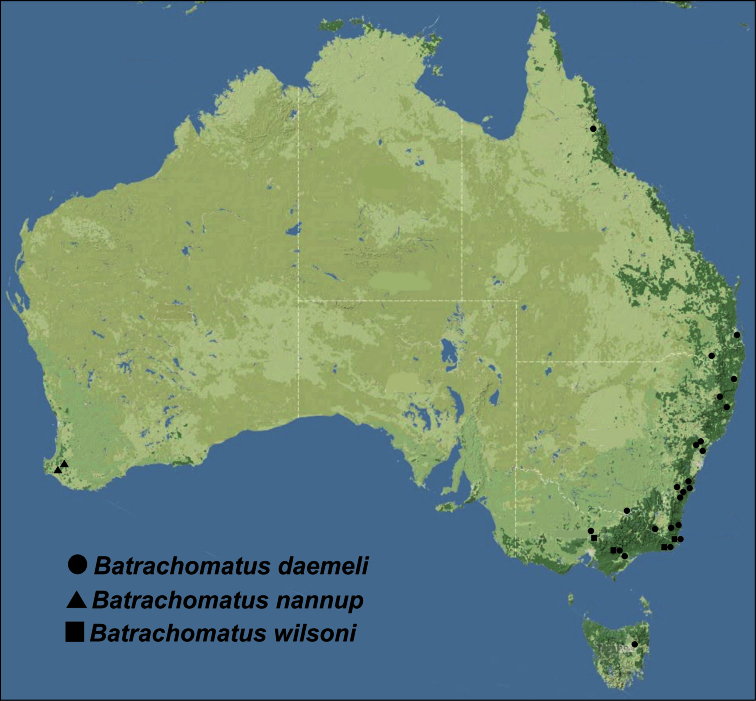
Distribution of *Batrachomatus*: *Batrachomatus daemeli* (dots), *Batrachomatus nannup* (triangles) and *Batrachomatus wilsoni* (squares).

**Figure 14.**
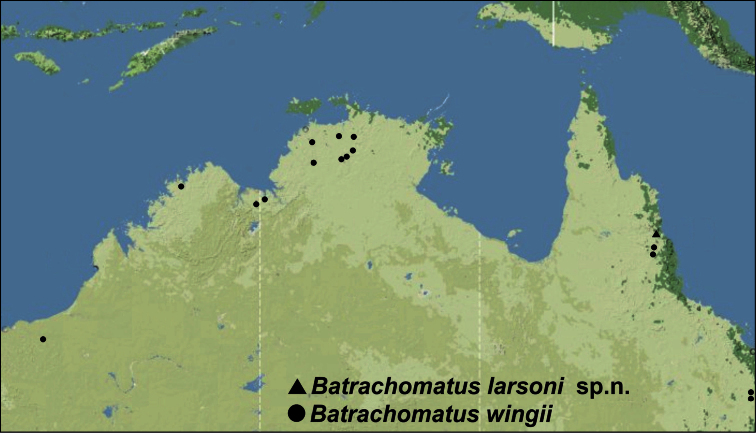
Distribution of *Batrachomatus*: *Batrachomatus larsoni* sp. n.(squares) and *Batrachomatus wingii* (dots).

**Figure 15.**
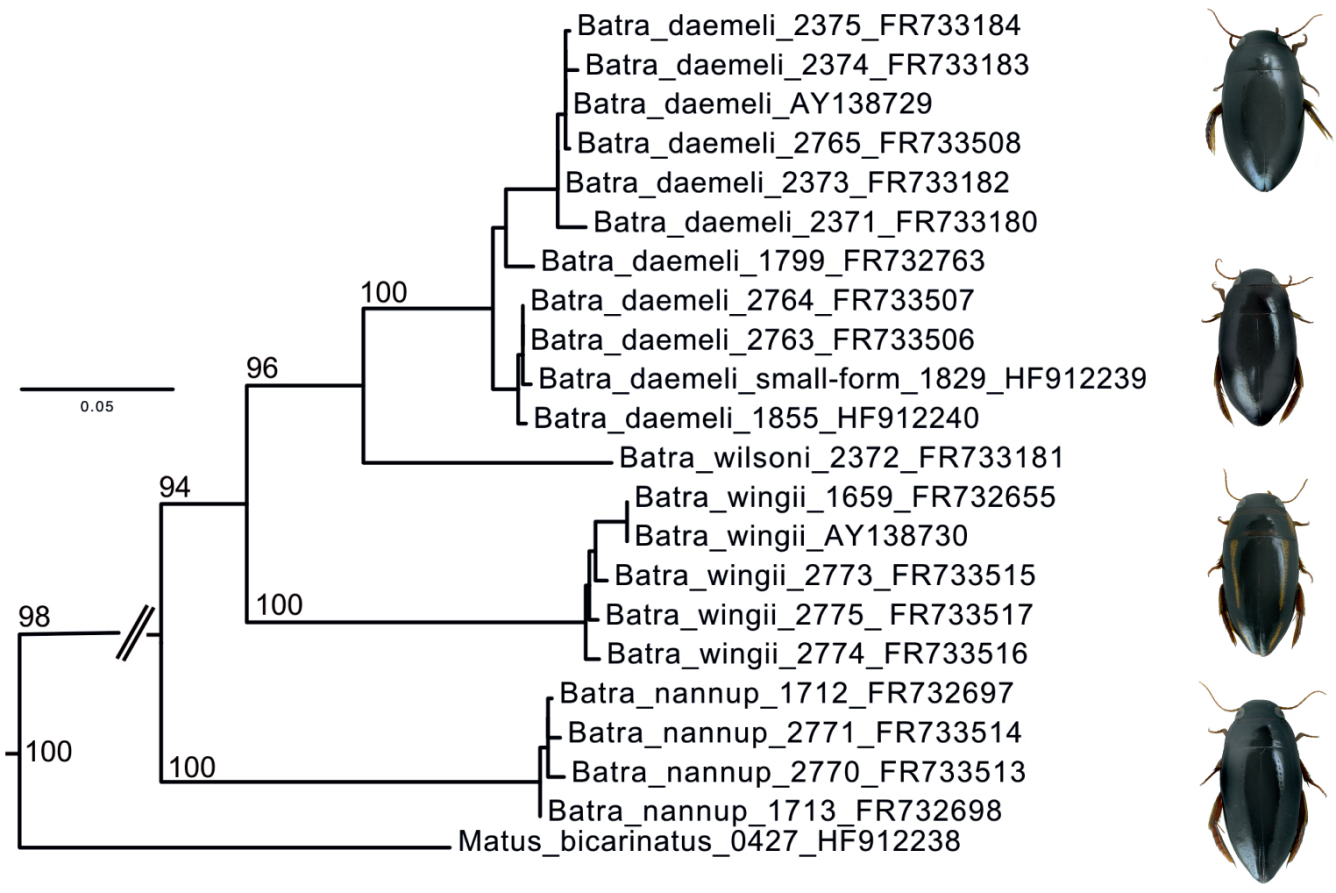
Maximum likelihood tree for Australian *Batrachomatus*. Node support are GARLI bootstrap values and only given for major nodes, numbers following taxon names are our extraction codes as well as Genbank accession numbers.

**Figures 16–21.**
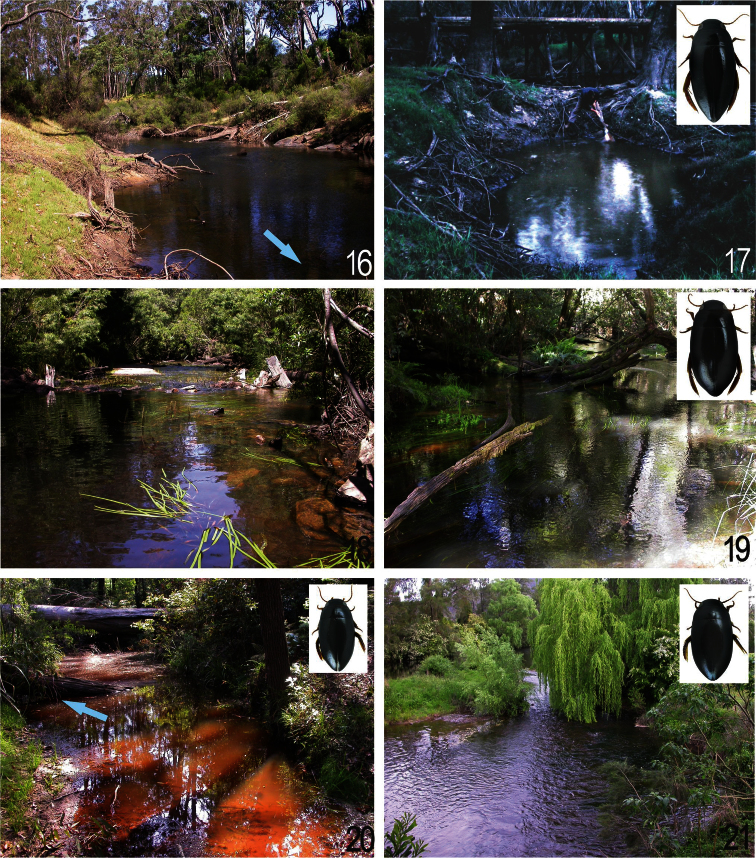
Habitats: **16, 17** Blackwood River upstream the village Nannup, habitat of *Batrachomatus nannup* in south-western Australia **18** Wallagaraugh River SW of Eden, southern New South Wales and **19** Thurra River at Highway 1 in south-eastern Victoria, habitats of *Batrachomatus wilsoni* **20** Coffs Harbour street, Flaggy Creek Nature Reserve, northern New South Wales and **21** Glouchestershire, Barrington River at Barrington, central New South Wales, habitats of *Batrachomatus daemeli* (Photos: L. Hendrich).

**Figures 22–27.**
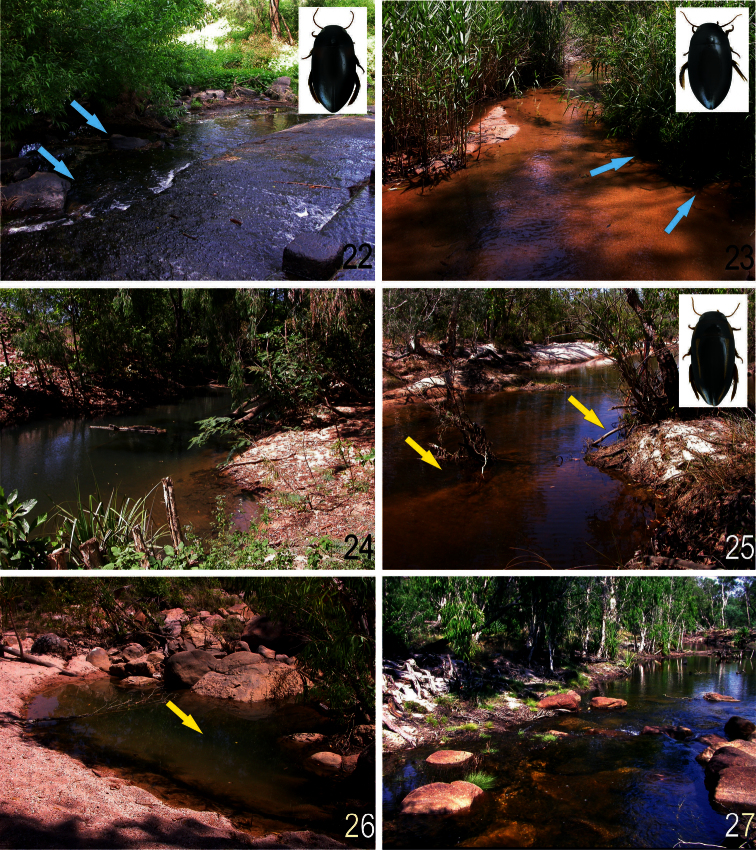
Habitats: **22** 3 km W Albion Park, North Macquarie Road at creek crossing, central New South Wales and **23** Hughes Creek at Avenel, Central Victoria, habitat of *Batrachomatus daemeli* **24** Finnis River, 10 km W Batchelor, Northern Territory **25** Magela Creek, Jabiru East, Northern Territory **26** Kakadu NP, Gunlom Waterfall Area, Northern Territory and **27** Kakadu N.P., Jim Jim District, Jim Jim Falls Camping Area, Jim Jim Creek, Northern Territory, habitats of *Batrachomatus wingii* (Photos: L. Hendrich).

### Batrachomatus daemeli

(Sharp, 1882)

http://species-id.net/wiki/Batrachomatus_daemeli

Matus daemeli Sharp, 1882: 600 (orig. descr.); Zimmermann 1920: 194 (cat.).
Batrachomatus daemeli (Sharp, 1882): Zimmermann 1919: 215 (comb. n.); Mouchamps 1964: 137 (descr.); Watts 1978: 119 (descr.); Watts 1985: 23 (cat.); Lawrence et al. 1987: 351 (cat.); Nilsson 2001: 261 (cat.); Watts 2002: 31, 46 (cat.).
Batrachomatus burnsi Mouchamps, 1964: 138; Watts 1978: 119 (comb. n.).
Batrachomatus burnsi var. *obscurior* Mouchamps, 1964: 140 (orig. descr.), **syn. n.**

#### Type locality.

Sydney, New South Wales, Australia.

#### Type material studied.

**Lectotype** ♂ **of *Matus daemeli***: “Lectotype” [Watts des. 1978: 119], “Type”, “Sydney Austr” [handwritten label], “S.Australia”, “Sharp Coll. 1905-313”, “Type 860 B. Daemeli” [handwritten label], “Batrachomatus daemeli Sydney” [handwritten label], “Matus daemeli Sharp Det. C. Watts 1979” (BMNH). **Holotype** ♂ **of *Batrachomatus burnsi***: “Macalister River, XI.1946, F.E.Wilson leg”, “Holotype” [red printed label], “Batrachomatus burnsi sp.n. Mouchamps” [handwritten and printed label] (MVMA).

#### Additional material studied

**(142 specimens)**: **Queensland**: 3 exs., “N QLD, Atherton Tableland, Millaa Millaa Falls, 2500 feet, IV.1932, Australia Harvard Exp. Darlington” (ANIC, SAMA); 1 ex., “S QLD, Brisbane, 1.I.1952, C.Oke leg.” (MVMA). **Australian Capital Territory**: 1 ex., “Canberra, I.1961, C.H.S. Watts leg.” (SAMA). **New South Wales**: 4 exs., “N NSW, 20 km NE Tenterfield, Boonoo Boonoo River Cross., 949m, 12.X.2006, 28°52.486S, 152°06.246E, L. & E. Hendrich leg.” (NSW 67) (CLH, ZSM); 21 exs., “N NSW, 35–40 km S Grafton, road [Orara Way] to Coffs Harbour, Flaggy Creek NR, 5m, 15.X.2006, 29.58.587S, 152.58.452E, L. & E. Hendrich leg. (NSW 77)”, one specimen with green printed label “DNA M.Balke 1829” (CLH, ZSM); 6 exs., “C NSW, Glouchestershire, Barrington River at Barrington, 133m, 18.X.2006, 31°58.225S, 151°54.160E, L. & E. Hendrich leg. (NSW 82)”, one specimen with green printed label “DNA M.Balke 1799” (CLH); 8 adults and 14 larvae (LA III), “C NSW, 3 km W Albion Park, North Macquarie Road at creek crossing, 19m, 30.X.2006, 34°34.337S, 150°43.456E, L. & E. Hendrich leg. (NSW 87)” (CLH); 8 exs., “C NSW, Endrick River at Braidwood Road, 554m, 1.XI.2006, 35°05.193S, 150°07.182E, L. & E. Hendrich leg. (NSW 94)” (CLH); 9 ex., “C NSW, 10 km W Braidwood, Shoalhaven River at Bombay Bridge, 628m, 2.XI.2006, 35°25.419S, 149°42.582E, L. & E. Hendrich leg. (NSW 96)” (CLH, ZSM); 4 exs., “S NSW, Wallagaraugh River Picnic Area, 43 km SW Eden, 54m, 17.XI.2006, 37.22.079S 149.43.073E, L. & E. Hendrich leg. (NSW 112)”, four specimens “DNA M.Balke 2371”, “DNA M.Balke 2373”, “DNA M.Balke 2374”, “DNA M.Balke 2375” (CLH, ZSM); 1 ex., “Williams River near Dungog, 27.XI.1996, C.H.S. Watts leg.” (SAMA); 5 exs., “5 km W Bombala, Saucey Creek, 18.I.1997, C.H.S. Watts leg.” (SAMA); 24 exs., “Eccleston, J. Hopson” (ANIC, SAMA); 2 ex., “N NSW, Caparra (Lots 72, 73, 148) 35 km NW Taree, 3.I.1990” (ANIC); 2 exs., “Kindee N.S.W Sept. 1934 H.J. Carter”, “Batrachomatus daemeli Shp. Det. C. Watts 1971”, “K 215108” (AMS); 1 ex., “N NSW, Kindee [Kindee Creek] 50 km W Port Macquarie, IX.1934” (ANIC); 1 ex., “C NSW, Shoalhaven River, 3.I.2001, W.D. Shepard leg.” (ANIC); 1 ex., “C NSW, Wollondilly R. Jooriland, 60 km W Wollongong, 16.XII.1931” (ANIC); 1 ex., “NSW EPA Survey MRHI SHOA (Shoalhaven River System) 01, Kangaroo River and Gerrigong Creek, 26.XI.1997, 34°41.17S, 150°35.58E J. Potts leg. K 227447 Riffle” (AMS); 1 ex., “NSW EPA Survey MRHI CLYD (Clyde River System) 13 d/s, Bimberamala River, 25.X.1995, 35°25.59S, 150°11.36E, A. Leask leg. EPA 08191 Edge” (AMS); 1 ex., “NSW EPA Survey MRHI SHOA 08 (Shoalhaven River System), Upper Corang River, 22.XI.1995, 35°12.19S, 150°03.24E, J. King leg., K 227320, edge” (AMS); 1 ex., “NSW EPA Survey MRHI HAST (Hastings River) 10 Forbes River: Round Flat, 2.X.1994, 31°20.20S, 152°20.23E, E. Turak leg., EPA 10992, logs” (AMS);8 exs., “S NSW, Albury, 16.VII.1989, P. Walker leg.” (SAMA). **Victoria**: “6 exs., E VIC, Thurra River at Hwy 1, Water Point Rest Area, 138m, 5.XI.2001, 37°34.061S, 149°16.338E, G.L.Challet leg.” (CGC, ZSM); 5 exs., “C VIC, Hughes Creek at Avenel, 161m, 25.XI.2006, 36°54.221S, 145°14.191E, L. & E. Hendrich leg. (VIC 120)”, one specimen with green, printed label “DNA M.Balke 2763”, “DNA M.Balke 2764”, “DNA M.Balke 2765” (CLH); 1 ex., “Western District Lakes Surv 78, 16.I.1980, W.D.Williams leg.” (SAMA); 1 ex., “Tamao Crossing, 24.I.1960, A.Neboiss leg.” (MVMA); 2 exs., “Maffra, I.1939, F.E.Wilson leg.” (MVMA). **Tasmania**: 1 ex., “Deloraine, Deloraine River, I.1961, C.H.S.Watts leg.” (SAMA).

#### Description.

**Measurements.** TL = 7.9–9.1 mm, TL-H = 7.3–8.2 mm; MW = 4.0–4.6 mm.

**Colour.** Dorsal surface shiny, head, pronotum and elytra black, legs dark reddish, antennae and palpi reddish pale. Some specimens with reddish basal markings on elytra (Figs 1, 2, 3).

**Structure and sculpture.** Body outline oblong oval. Dorsal surface densely and evenly covered with small punctures, and with a very fine, almost subobsolete close reticulation with large meshes. Serial punctures on elytron distinct, large and shallow. Ventral surface finely and densely punctate. Prothoracic process broad, flat, apex pointed, broadly grooved in middle for whole length, sides strongly margined. Metacoxal lines raised, well separated, reaching almost to hind margin of metaventrite, diverging a little anteriorly.

**Male.** Pro- and mesotarsi dilated and stouter than in female, furnished beneath with dense, short, stout setae arranged in groups, many of the setae ending in minute suction cups. Aedeagus: median lobe in lateral (Figs 8a) and in ventral view (Figs 8b); paramere (Fig. 8c).

**Variability.** A very variable species in size and colour. Specimens having reddish shoulders of various form and extension were named *Batrachomatus burnsi* Mouchamps, 1964 (Fig. 2, large red patch on the shoulder) and *Batrachomatus burnsi* var. *obscurior* (smaller and interrupted reddish patch on shoulder). Such specimens can be found all over the species distributional range.

According to our study of the complete material of *Batrachomatus daemeli* two different forms can be recognized: larger specimens (see measurements above) with larger median lobes and smaller specimens [Measurements: TL = 7.3–8.0 mm, TL-H = 6.5–7.05 mm; MW = 3.6–3.9 mm] (Fig. 3) with smaller and less elongated median lobes. The latter have been collected at several places in New South Wales and Victoria, sometimes syntopic with the larger form. Despite the fact that intergrades between the typical *Batrachomatus daemeli* and the “smaller form” have been found, for some time we even have been strongly tempted to describe those smaller specimens as another new species. On the other hand we have not been able to find any constant extern morphological character—except of the size—and also the shapes of the male genitalia are more or less the same. In addition, in our c*ox1* tree (Fig. 15) specimens of both forms aregrouped in the same clade.

#### Affinities.

*Batrachomatus daemeli* differs from the northern Australian *Batrachomatus larsoni* sp. n. and *Batrachomatus wingii* in the broader, more oblong shape, the lack of colour pattern on upper surface in most specimens, the weakly diverging metacoxal lines and the shape of the median lobe.

#### Distribution.

The most widespread and common species of the genus in Australia (Fig. 13). From the Atherton Tableland in Queensland along the east coast of New South Wales, Canberra area, to western Victoria and north-eastern Tasmania (Watts 1978).

#### Habitat.

*Batrachomatus daemeli* inhabits permanent streams, creeks and slow flowing larger rivers at an altitude from about sea level to almost 1000 m, from more or less open country in cultivated areas to closed-canopy forest sites (Figs 20–23). Most specimens were found in low-gradient stream or river sections where the substrate was enriched with rotten leaves, wood and larger stones. In this habitat the beetles were found in areas of medium, laminar flow, generally in deeper water (50 cm depth and more) under larger logs and stones. When disturbed the adults can be observed swimming around and coming to the surface. In dryer periods the species can be found in the deepest parts of the remaining rest pools of a creek or river. All the mentioned smaller specimens from Coffs Harbour, Flaggy Creek were collected in a peaty and intermittent creek, with muddy bottom, shaded by wet eucalypt forest. The adults were mainly being located in a mixture of water and mud under a larger rotten tree trunk (Fig. 20). At this site *Batrachomatus daemeli* was associated with the rarely collected *Sternopriscus wallumphilia* Hendrich & Watts, 2004, a dytiscid known before only from its type locality, a single creek in the Wallum heath near Glasshouse Mountains in southern Queensland (Hendrich and Watts 2004). In New South Wales the larvae of *Batrachomatus daemeli* were collected together with the adults in October and November. Adults can be found all over the year.

### Batrachomatus larsoni sp. n.

urn:lsid:zoobank.org:act:D753FBD7-A28A-4AA5-A6A8-5FF35E0B3495

http://species-id.net/wiki/Batrachomatus_larsoni

Allomatus new species: Larson 1993: 49 (cat.).

#### Type locality.

Creek, Windsor Tableland access road [16°13'55"S, 145°1'27"E], Queensland, Australia.

#### Type material.

**Holotype** ♂: “AUSTRALIA, QLD Windsor Tableland access rd km 40 Nov. 12/90 Larson” [white printed label], “Holotype Batrachomatus larsoni sp.n. Hendrich & Balke des. 2010” [red printed label] (ANIC). **Paratype** ♀ with same data as holotype (ANIC). The single paratype is provided with a red printed paratype label.

#### Description. 

**Measurements.** Holotype: TL = 7.9 mm, TL-H = 7.1 mm; MW = 3.85 mm. Paratype: TL = 7.7 mm, TL-H = 7.0 mm; MW = 3.8 mm.

**Colour.** Dorsal surface shiny, black, appendages reddish.Head black with epistome and labrum lighter. Pronotum with reddish broad lateral margin. Elytron with narrow reddish band along 2/3 of length of elytron (Fig. 4).

**Structure and sculpture.** Body outline oblong oval, only slightly convex. Head, pronotal and elytral surface covered by polygonal double reticulation, smaller superficial meshes inside larger and more visible meshes, with punctures at intersections of all the larger meshes. Sides of pronotum moderately curved and convergent anteriorly. Sculpture on elytra as in pronotum but punctures at the intersections of all larger meshes smaller. Serial punctures on elytron distinct, large and shallow. On ventral side, metacoxal plate with very fine microreticulation, meshes very elongate, inside minutely and sparsely punctate. Lateral wings of metaventrite very narrow. Prosternal process flat, broad, broadly carinate in midline, parallel-sided, weakly pointed apically, weakly margined. Metacoxal lines well separated, strongly diverging anteriorly. Area between metacoxal lines with many small punctures.

**Male.** Pro- and mesotarsus a little dilated, basal 3 tarsomeres with dense short setae beneath suction cups. Aedeagus: median lobe (Fig. 9a, b); paramere (Fig. 9c).

#### Etymology.

This species is dedicated to our Canadian colleague David Larson (Maple Creek, Canada) who collected the only known specimens and recognized the species as new. The specific epithet is a substantive in the genitive case.

#### Affinities.

*Batrachomatus larsoni* sp. n. differs from *Batrachomatus wilsoni* by its smaller size (*Batrachomatus larsoni* TL = 7.9 mmand *Batrachomatus wilsoni* TL = 8.4–8.5 mm), in the lack of any reddish humeral angles on elytra, the more flattened and narrowly formed body, and in having the reticulation on the elytra weak without punctuation, instead of moderately strong and punctate. Both species can be separated by the shape of their median lobes.

#### Distribution.

Only known from the type locality in NE Queensland (Fig. 14).

#### Habitat.

The Windsor Tableland is a granite plateau at about 1100 m, near to but further inland from Mt. Spurgeon and Mt. Lewis, in north-east Queensland. Because of its altitude, it receives enough rainfall to sustain mountain rainforest over much of the plateau surface, although it is surrounded by tropical eucalypt savannah at lower altitudes. Access to the Windsor Tableland is now for scientific study only, the public are permanently barred. A very detailed habitat description is given by Larson (1993): “Two specimens were collected from a low gradient section of a small, permanent, closed forest stream. The stream was largely shaded by a tall, more or less closed tree canopy. The stream bed was coarse sand and consisted of shallow, gentle riffles which separated pools formed where sand had been scoured from around and behind logs and from under tree roots to produce pools under overhanging roots-mats. The specimens were found swimming in a pool after the trailing roots on an overhanging bank on one side of the pool had been vigorously swept with a net. It is assumed the beetles came from under the bank but similar habitat, which was common along the stream, was searched without yielding additional specimens”.

### Batrachomatus nannup

(Watts, 1978) comb. n.

http://species-id.net/wiki/Batrachomatus_nannup

Allomatus nannup Watts, 1978: 117 (orig. descr.); Watts 1985: 23 (cat.); Lawrence et al. 1987: 351 (cat.); Nilsson 2001: 261 (cat.); Watts 2002: 30, 46 (cat.).

#### Type locality.

Bridgetown [Blackwood River, 33°58'02"S, 116°07'58"E], Western Australia, Australia.

#### Type material.

**Holotype** ♂: “Bridgetown Nov 8 31 W.A.” [handwritten label], “Australia Harvard Exp. Darlington” [printed label], “Holotype Allomatus nannup Det. C. Watts 1976” [handwritten and printed label] (ANIC). **Paratype** ♂: “Bridgetown Nov 8 31 W.A.” [handwritten label], “Australia Harvard Exp. Darlington” [printed label], “Paratype Allomatus nannup Det. C. Watts 1976” [handwritten and printed label], “SAMA Database No. 25-006714” [printed label] (SAMA).

#### Additional material 

**(178 specimens)**: **Western Australia**: 2 exs., “Nannup-Balingup Road, Blackwood River, 28.IX.1965, E.B. Britton leg.” (ANIC); 57 exs., “Blackwood River near Nannup, 20.X.1996, C.H.S. Watts” (SAMA); 8 exs., “Blackwood River near Nannup, 20.IX.2000, C.H.S. Watts” (SAMA); 1 ex., “Blackwood River near Bridgetown, 21.IX.2000, C.H.S. Watts” (SAMA); 20 exs., “Nannup, Balingup-Nannup Road, Blackwood River at Revelly Bridge, 79m, 33.55.226S, 115.48.549E, 25.XI.1996, L. Hendrich leg. (Lok. 33)” (CLH, ZSM); 65 exs., “Blackwood River, 8.3 Km NE Nannup, Revelly Bridge, 79m, 6.I.2007, 33.55.226S, 115.48.549E, L. & E. Hendrich leg. (WA 166)”, four specimens provided with green printed labels “DNA M.Balke 1712”, “DNA M.Balke 1713”, “DNA M.Balke 2771”, “DNA M.Balke 2772” (BMNH, NMW, ZSM); 25 exs., “Nannup, Balingup-Nannup Road, Revelly Bridge, 79m, 31.XII.1999, 33.55.226S, 115.48.549E, Hendrich leg. (Loc. WA 6/153)” (CLH, ZSM).

#### Description. 

**Measurements.** TL = 9.1–9.6 mm, TL-H = 8.2–8.6 mm; MW = 4.0–4.3 mm.

**Colour.** Dorsal surface shiny, black; appendages reddish. Head black with epistome and labrum lighter (Fig. 5).

**Structure and sculpture.** Body outline narrowly oval, flattened and only slightly convex, pointed apically. Head with strongly impressed reticulation, meshes moderately large and irregular, some scattered small punctures; reticulation and punctures in pronotum rather weak. Elytron with fine reticulation, with transversely elongate small meshes, virtually impunctate apart from serial punctures, being comparatively large and well marked.

Ventral side with metacoxal plate covered by very fine microreticulation, meshes very elongate, inside minutely and sparsely provided with small punctures. Prosternal process flat, broad, broadly carinate in midline, parallel-sided, weakly pointed apically, weakly margined. Metacoxal lines well separated, subparallel in anterior 2/3, moderately diverging posteriad, area between lines with many small punctures.

**Male.** Pro- and mesotarsi a little dilated, basal 3 tarsomeres with dense short setae beneath suction cups. Aedeagus: median lobe (Fig. 10 a, b); paramere (Fig. 10 c).

#### Affinities.

*Batrachomatus nannup* differs from most specimens of *Batrachomatus wilsoni* and *Batrachomatus larsoni* sp. n. in the lack of any reddish humeral angles to elytra, the more flattened and narrowly formed body, and in having the reticulation on the elytra weak without punctuation, instead of moderately strong and punctuate. All three species can be easily separated by the shape of their median lobes and their distribution.

#### Distribution.

An endemic species of the Blackwood River in south-western Australia. All records between Bridgetown and Nannup but probably more widespread in the Blackwood River and its larger tributaries (Fig. 13).

#### Habitat.

The Blackwood River is the largest river in south-western Australia. The river begins near Quelarup and runs in a south-western direction through the town of Bridgetown then through Nannup until it discharges into the Southern Ocean at Hardy Inlet near the town of Augusta. The river has 41 tributaries and the upper or larger catchment area of the river is in agricultural areas, while the middle catchment area passes through forested areas, and the lower portion of the river passes into mixed forest, agricultural and residential lands (Wikipedia).

At the sampling localities the Blackwood is partly shaded by old River gum trees. In late spring the adults of *Batrachomatus nannup* can be either found in larger (6–10 m²) and deeper (40–80 cm depth) sandy pools in the floodzone of the river or among floating roots, rotten twigs and logs in shallow water of protected embayments of the slow flowing river (Fig. 16). In summer and in dryer periods the adults were collected only in the deepest parts of the almost standing river, under larger logs, stones and rotten debris (Fig. 17). When disturbed the beetles can be observed swimming around and coming to the surface.

### Batrachomatus wilsoni

(Mouchamps, 1964) comb. n.

http://species-id.net/wiki/Batrachomatus_wilsoni

Allomatus wilsoni Mouchamps, 1964: 140 (orig. descr.); Watts 1978: 116 (descr.); Watts 1985: 23 (cat.); Lawrence et al. 1987: 351 (cat.); Nilsson 2001: 261 (cat.); Watts 2002: 30, 46 (cat.).

#### Type locality.

Kerrisdale [King Parrot Creek, 165 m, 37°8'8"S, 145°15'36"E], Victoria, Australia.

#### Type material.

Holotype ♂: Not seen. **Paratype** ♂: “Macalister Riv 11/46 Vic F.E.Wilson”, “Wilson Coll” [handwritten label by Mouchamps], “Paratype” [red, printed label], “Paratype 3804” [blue printed label], “R.Mouchamps det., Allomatus wilsoni nsp.” [handwritten, white label by Mouchamps] (MVMA).

#### Additional material

**(2 specimens)**: **New South Wales**: 1 ex., “S NSW, Wallagaraugh River Picnic Area, 43 km SW Eden, 54m, 17.XI.2006, 37.22.079S 149.43.073E, L. & E. Hendrich leg. (NSW 112)”, “DNA M.Balke 2372” [green printed label] (ZSM). **Victoria**: 1 ex., “E VIC, Thurra River at Hwy 1, Water Point Rest Area, 138m, 17.XI.2006, 37.34.061S, 149.16.338E, L. & E. Hendrich leg. (VIC 114)” (CLH).

#### Description. 

**Measurements.** TL = 8.4–8.5 mm, TL-H = 7.5–7.7 mm; MW = 4.0–4.15 mm.

**Colour.** Head black with epistome and labrum lighter. Palpi and antennae brownish. Pronotum entirely black, slightly lighter at margins. Elytra completely black or with more or less developed brownish humeral patch. Ventral side black, appendages lighter with tarsi reddish brown (Fig. 6).

**Structure and sculpture.** Body outline oval, large, slightly convex. Head with anterior border of epistome a little excavated, not bordered. Head and pronotum surface covered by polygonal double reticulation, smaller superficial meshes inside bigger meshes, with punctures at intersections of all bigger meshes. Sides of pronotum moderately curved and convergent anteriorly. Sculpture on elytra consisting of a weak double reticulation, meshes polygonal, large, smaller and very fine punctures at intersections of very few meshes. Serial punctures on elytra distinct, large and shallow. Ventral surface covered with weakly defined reticulation, meshes, very elongate and more or less oblique or transverse. Prosternal process flat and excavated in anterior midline by median groove. Lateral borders of prosternal process clearly raised. Lateral wings of metaventrite narrow. Metacoxal lines separating metacoxal plate into three unequal parts; the median sparsely punctured, the lateral and the intralinear space almost smooth with an extremely sparse and scarcely visible microreticulation.

**Male.** Pro- and mesotarsi dilated and stouter than in female, furnished beneath with dense, short, stout setae arranged in groups, many of the setae ending in minute suction cups. Aedeagus: median lobe (Fig. 11 a, b); paramere (Fig. 11 c).

#### Affinities.

*Batrachomatus wilsoni* isin body outline and coloration very near to *Batrachomatus daemeli* but can be easily separated by the presence of a superficial polygonal double reticulation on pronotum and elytra. *Batrachomatus wilsoni* differs from *Batrachomatus larsoni* sp. n. by its larger size (*Batrachomatus wilsoni* TL = 8.4–8.5 mm and *Batrachomatus larsoni* TL = 7.9 mm), the different elytral coloration, and the more oblong and less narrowly formed body. Furthermore, all three species can be separated by the shape of their median lobes.

#### Distribution.

South-eastern Australia (Fig. 13). A rarely collected species with a very limited distribution. Only known from four sites from southern New South Wales (Wallagaraugh River) to southern and south-eastern Victoria (Macalister River, King Parrot Creek, Thurra River).

#### Habitat.

*Batrachomatus wilsoni* inhabits permanent slow flowing larger rivers, at an altitude from about sea level to almost 170 m, in closed-canopy old growth forest sites. The type locality King Parrot Creek, near Kerrisdale, is in many parts a low gradient river, similar to the south-western Australian Blackwood River. The two recently collected specimens from Victoria and New South Wales were found in low-gradient river sections where the substrate was enriched with rotten leaves, wood and larger stones (Figs 18, 19). In this habitat the beetles were found in areas of medium, laminar flow, generally in deeper water (50 cm and more) under larger logs and stones, always together with numerous *Batrachomatus daemeli*.

### Batrachomatus wingii

Clark, 1863

http://species-id.net/wiki/Batrachomatus_wingii

Batrachomatus wingii Clark, 1863: 15 (orig. descr.); Mouchamps 1964: 138 (descr.); Watts 1978: 120 (descr.); Watts 1985: 23 (cat.); Lawrence et al. 1987: 351 (cat.); Larson 1993: 50 (cat.); Nilsson 2001: 261 (cat.); Watts 2002: 31, 46 (cat.).

#### Type locality.

Northeast coast of Australia.

#### Holotype

♂: “N. Holl NE Aust 4412” [handwritten label], “Holotype”, “Batrachomatus wingi” [sic!], [blue handwritten label by Clark] (BMNH).

#### Additional materia

**l (71 specimens).** **Western Australia**: 1 ex., “Pilbara Region, De Grey River at Yarrie Station, 10.VII.1953, N.B.Tindale leg.” (SAMA); 1 ex., “East Kimberley, Mitchell Plateau, Surveyors Pool, 150m, 17.VI.1999, Hendrich leg.” (CLH). **Northern Territory**: 18 exs., “Kakadu N.P., Gungurul Lookout, 50 m, 13.59.359S, 132.19.904E, 1.XI.1996, L. Hendrich leg. (Lok.11)” (CLH); 3 exs., “Finnis River, 10 km W Batchelor, 43m, 20.VIII.2006, 13.01.278S, 130.57.217E, L. & E. Hendrich leg.” (NT 2), provided with green printed labels “DNA M.Balke 2773”, “DNA M.Balke 2774”, “DNA M.Balke 2775” (ZSM, CLH); 4 exs., “Kakadu NP, Gunlom Waterfall Area, 72m, 25.VIII.2006, 13.26.026S, 132.25.141E, L. & E. Hendrich leg.” (NT 17), one specimen with green printed label “DNA M.Balke 1659” (ZSM); 20 exs., “Magela Creek upstream, Jabiru East, 38m, 29.VIII.2006, 12.40.458S,132.55.853E, L. & E. Hendrich leg.” (NT 21) (CLH, NMW, ZSM); 1 ex., “Magela Creek downstream, Jabiru East, 31m, 30.VIII.2006, 12.38.312S, 132.53.441E, L. & E. Hendrich leg. (NT 23)”, “DNA M.Balke 2772” [green printed label] (ZSM); 1 ex., “Daly River, H. Wesselmann” (SAMA); 4 exs., “Kakadu N.P., Jim Jim District, Jim Jim Falls Camping Area, Jim Jim Creek, 60 m, 13.16.218S, 132.49.276E, low-gradient stream, 26. & 27.X.1996, Hendrich leg./loc. 2a” (CLH);1 ex., “Kakadu N.P., Mary River District, 3 km ESE Gunlom Camping Area, South Alligator River, 50m, 2.XI.1996, 13.27.276S, 132.26.268E, L. Hendrich leg.” (loc. 14) (CLH); 1 ex., “Fergusson River, 31 km SE by S of Pine Creek, 14.XI.1979, T.A.Weir leg.” (ANIC). **Queensland**: 6 exs., “Foleyvale Aboriginal Reserve, 130 km W Rockhampton, 20.–25.I.1968, G. Hangay leg.” (HNHM, CLH); 4 exs., “Boolburra, 95 km WSW Rockhampton, 12.I.1968, G. Hangay leg.” (HNHM, CLH); 1 ex., “N Queensland, Bridge Creek, 20.XI.1992, water sweep, A. Calder & P. Zborowski leg.” (ANIC); 1 ex., “N Queensland, Kennedy River Xing, dry river bed, sandy base, temporary pool, 16.VI.1992, T.A. Weir leg.” (ANIC). Without any detailed locality label: 4 exs., “Australien”, “Sammlung Clemens Müller”, “Sammlung Zimmermann” (ZSM).

**Literature records**: Australia, Western Australia, Weaber Plain, Keep River east of Milligans, 15°37'11"S, 129°02'00"E, May 2009, A.W. Storey leg. (WRM 2010); Australia, Northern Territory, Sandy Creek downstream of Keep River at Legune Road Crossing, 23.VII.–1.VIII.2004, 15°22'54"S, 129°11'43"E, A.W. Storey leg., site code SE 1, idem, Sandy Creek upstream of Keep River Road, 23.VII.–1.VIII.2004, 15°24'30"S, 129°11'33"E, A.W. Storey leg., site code SR 2 (NCTWR 2005).

#### Description. 

**Measurements.** TL = 6.9–7.8 mm, TL-H = 6.2–7.2 mm; MW = 3.2–3.7 mm.

**Colour.** Black, anterior part of head reddish brown, pronotum with broad yellow lateral margins, elytron with a narrow yellow band of which apical 1/3 close to side and basal 2/3 some distance from side (Fig. 7). Underside and parts of head reddish brown,appendages reddish brown.

**Structure and sculpture.** Body outline elongate oval, flattened, pointed apically. Dorsal surface shiny, head, pronotum and elytral surface densely and evenly covered with small punctures, reticulation absent. Serial punctures on elytron sparse, weakly impressed, indistinct. Sides of pronotum moderately curved and convergent anteriorly. Ventral surface very densely and minutely punctured. Prosternal process flat, broad, parallel-sided, weakly and narrowly grooved in midline, and weakly margined at side, tip bluntly pointed, apex pointed. Metacoxal lines well separated, subparallel, reaching almost to hind margin of metaventrite.

**Male.** Pro- and mesotarsi stouter than in female, furnished beneath with dense, short, stout setae arranged in groups, many of the setae ending in minute suction cups. Aedeagus: median lobe (Fig. 12 a, b); paramere (Fig. 12 c).

#### Affinities.

A very characteristic species and one of the most beautiful dytiscids in Australia (Fig. 7). *Batrachomatus wingii* differs from all other species of the genus in the narrower and more flattened shape of body, the dorsal yellowish longitudinal stripes on the elytra, the subparallel metacoxal lines and the shape of the median lobe.

#### Distribution.

Tropical northern Australia. Occurring from the northern Pilbara and the Kimberley region in the northwest, the Daly River, Darwin area and Kakadu National Park in the north, to the Atherton Tableland in northern Queensland, and along the east coast south to Rockhampton (Fig. 14).

#### Habitat.

*Batrachomatus wingii* occurs in seasonal and permanent lowland streams, creeks and slow flowing smaller rivers at an altitude from about 20 to almost 150 m, at least partly shaded by eucalypt woodland or monsoonal forest. Most specimens were found in low-gradient stream sections where the substratum was entirely coarse sand and smaller pebbles yet the current was strong enough to clear the bottom of silt and leaves (Figs 24, 25, 27). In this habitat the beetles were found in areas of medium, laminar flow, generally in deeper water (50 cm depth and more), along the outside curve of stream bend, among floating gum roots, under larger logs and stones. In the Northern Territory, at the end of the dry season, a larger series of the species was collected in the deepest, coldest and most oxygen rich part (50–80 cm depths) of a rest pool (10 m²), situated in a broad and almost dry and sandy creek bed. The pool was without any vegetation but rich in rotten leaves and partly shaded (Fig. 26). As mentioned by Larson (1993), who collected the species in northern Queensland at three different sites, the sub-surface seepage and local water temperatures could also be factors responsible for the local aggregation of beetles. In the Northern Territory larvae have been collected after the rainy season in February by Watts (Alarie et al. 2001).

##### Habitats and faunistics

All Australian *Batrachomatus* species are strongly lotic and restricted to streams, creeks and rivers with sand, pebble and cobble beds, often situated in woodland or closed-canopy forest sites (Figs 16–27). It is probable that at least the larvae are sensitive to low levels of dissolved oxygen and require cooler temperatures. If this is so the occurrence of any *Batrachomatus* species can be considered a good indicator of a running water´s health, habitat and water quality.

The currently known altitudinal distribution and ecology of *Batrachomatus* species is shown in Table 1. Most species occur in lowland regions from almost sea level to 500 m. Only *Batrachomatus daemeli* has been collected in the Atherton Tableland and the Great Dividing Range up to at least 950 m. In Queensland *Batrachomatus daemeli*, *Batrachomatus wingii* and *Batrachomatus larsoni* sp. n.are sympatric. In eastern Victoria *Batrachomatus daemeli* and *Batrachomatus wilsoni* are known from the same area and in three rivers they are also syntopic. *Batrachomatus daemeli* is widespread along the east coast and south-eastern Australia, including the northeast of Tasmania, and *Batrachomatus wingii* occurs all over the tropical north of Australia. Two species, *Batrachomatus nannup* in the south west and *Batrachomatus larsoni* sp. n. in NE Queensland, have a very restricted distribution and are only known from one stream or river system (Blackwood River, Windsor Tableland). The rarity and limited distribution of the south-eastern *Batrachomatus wilsoni* might be the result of human impacts (irrigation, increasing of salinity, clearing of riverine forests) on almost all river systems in Victoria in the last five decades. All species seem to be capable of flight but none was ever obtained by operating light traps. The larvae of *Batrachomatus daemeli* and *Batrachomatus nannup* were recently described by Alarie et al. (2001). The larvae of *Batrachomatus wilsoni*, *Batrachomatus larsoni* sp. n. and *Batrachomatus wingii* remain unknown.

When in a net and out of the water specimensof *Batrachomatus* move very rapidly and are easily to recognise and to collect. In contrast, Larson et al. (2000) noted that the related Nearctic *Matus* are all lentic and occur along the margins of rather eutrophic ponds, often amongst *Typha* or decaying deciduous leaves. They are often slow to move and with their brownish colour are easily overlooked and consequently are not well collected. Despite the fact that all Australian species are capable of flight, none was obtained by operating light traps.

**Table 1.** Habitat information and altitudinal distribution of Matiniin Australia.<br/>

**Species**	**Altitude**	**Habitat**
*Batrachomatus daemeli*	5–950 m	Large rivers, creeks and streams, side pools
*Batrachomatus larsoni* sp. n.	500 m	Small rainforest stream
*Batrachomatus nannup*	79 m	Large permanent river and side pools (adults)
*Batrachomatus wilsoni*	54–138 m	Larger permanent rivers
*Batrachomatus wingii*	30–150 m	Seasonal and permanent rivers, streams and creeks

##### Key to *Batrochomatus*

**Table:** 

1	Dorsal surface covered with a strong double reticulation of irregular and polygonal meshes. Punctures absent or only at the intersections of all larger meshes	2
–	Dorsal surface densely and evenly covered with small punctures, reticulation absent or very fine almost obsolete with larger meshes	4
2	Pronotum totally black	3
–	Pronotum black, with reddish broad lateral margins. Dorsal surface covered by a polygonal double reticulation with punctures at the intersections of all larger meshes. Elytra with a narrow longitudinal reddish band	*Batrachomatus larsoni* sp. n. (NE Queensl.)
3	Body outline oval, slightly convex. Humeral angle of elytron reddish. Punctures at the intersections of a very few larger meshes. Metacoxal lines separating metacoxal plate into three unequal parts; the median sparsely punctured, the lateral almost smooth with an extremely sparse and scarcely visible microreticulation. Aedeagus: median lobe (Fig. 11 a, b)	*Batrachomatus wilsoni* (SE Australia)
–	Body outline narrowly oval, flattened and only slightly convex, pointed apically. Elytron uniformly black, reticulation virtually impunctate. Metacoxal lines well separated, subparallel in anterior 2/3, moderately diverging posteriad, area between lines with many small punctures. Aedeagus: median lobe (Fig. 10 a, b)	*Batrachomatus nannup* (SW Australia)
4	Pronotum totally black. Elytra with or without a red humeral angle. Body outline oblong oval. Dorsal surface with a very fine almost obsolete close reticulation with larger meshes. Metacoxal lines raised, well separated, reaching almost to hind margin of metaventrite, diverging a little anteriorly. Aedeagus: median lobe (Fig. 8 a, b)	*Batrachomatus daemeli* (SE Australia)
–	Pronotum with broad yellow lateral margins, elytron with a narrow yellow band of which apical 1/3 is close to side and basal 2/3 some distance from side. Body outline elongate oval, flattened, pointed apically. Reticulation on dorsal surface absent. Metacoxal lines subparallel. Aedeagus: median lobe (Fig. 12 a, b)	*Batrachomatus wingii* (N. Australia)

## Supplementary Material

XML Treatment for Batrachomatus

XML Treatment for Batrachomatus daemeli

XML Treatment for Batrachomatus larsoni

XML Treatment for Batrachomatus nannup

XML Treatment for Batrachomatus wilsoni

XML Treatment for Batrachomatus wingii
